# The Exceeding Importance of a Clear Understanding of the Vasomotors and the Utilization of Their Function to Get Best Therapeutic Results

**Published:** 1907-10

**Authors:** W. C. Abbott

**Affiliations:** Chicago, Ill.


### The Exceeding Importance of a Clear Understanding of the Vasomotors and the Utilization of Their Function to Get Best Therapeutic Results.

BY W. C. ABBOTT, M. D., CHICAGO, ILL.

Whatever may be the anatomical structure that affords the phenomena we term vasomotor, it is certain that perturbations of vasomotor equilibrium form the earliest evidences of disease in the vast majority of maladies. So delicate is the adjustment of the governing apparatus that the first evidence of approaching disease is almost invariably to be displayed in alterations in the vascular tension of some or other area. Let this be due to the advent of an invading swarm of bacteria, or to the presence of a chemic irritant in the blood conveyed to the part, the first response to any abnormal irritation is to be seen in an alteration of the caliber of some part of the circulatory tract; in fact, such a change is usually requisite for the production of pathological lesions that are recognizable by microscopic investigation.

In venules, arterioles or capillaries, or in all three, we have first either a condition of spasm by which the vessels are contracted and the blood is forced out into the general circulation, or a state of paresis, the vessels dilated and containing more than the normal quantity of blood which leaks into the area of low pressure from the general circulatory reservoir. Here commences the anatomy of disease—and that frightfully neglected process, the physiology of disease. Too much attention and too much importance have been given to the dead tissues, too little to the aberrations from normality displayed by the still living organs and acting functions. It is with living, functionating tissues that we have to deal; with pathological processes while still active, in bodies not yet devitalized, and not with the final end-results of these processes in bodies that have succumbed. If a patient is still alive today, he should have enough vitality to keep him living for a succession of tomorrows, provided we can successfully cope with his malady and prevent its making further progress. We may, then, take up the question of eradicating the malady, which is another matter.

The physician who accustoms himself to study the conditions presented by the vasomotors soon learns to detect the earlier perturbations, to recognize local "jangles," and to apply his remedies before the difficulty has progressed beyond the simple disorder of tonicity-excess or deficiency. It may not be easy to give a nosological designation to these cases, and the physician may have his own opinion as to whether in dissipating the disease and restoring circulatory equilibrium he has aborted a pneumonia or jugulated any other affection; but he knows that just such cases let alone do develop into maladies that can, without the slightest difficulty, be recognized and classified, and that in their fully developed forms may really verify the pessimistic dictum—there is no treatment that cures.

How many diseases of the body politic could be cured in their incipiency by a little, a very little common sense? How many destructive wars could have been checked at the outset if only the principals had had a little more mutual consideration? How many floods could have been prevented had there but been a boy along to stop the beginning crevasse in the levee with a chunk of sod? Even the case of tetanus or of rabies might have been jugulated by a prompt excision of the virulent microbic colony at the start, to say nothing of the ease with which most acute affections may be jugulated. Innumerable similar instances could be adduced showing the efficacy of well-placed intervention at the beginning of morbid processes.

It will be objected that this treatment of a symptom is unscientific, in that it does not strike at the cause of the illness, but is aimed at a result, and a superficial one at that. This is another of those illogical jumpings at conclusions without warrant which we have so often cited. How do you know the treatment that removes a symptom does not remove its cause? Is it not *prima facie* evidence that it does so when the results disappear? Take any symptom-complex that presents itself, it evidently depends on some pathological aberration, but just what we are, in the vast majority of cases, unable to say. But whatever it may be, it is a fair inference that the treatment that restores to health, or that causes the disappearance of that symptom must remove the cause—otherwise, if the cause is still active, why does not the symptom remain? This, of course, does not apply to the smothering of pain by morphine or of fever by cold, although there are not wanting those who claim actual curative action from these measures, and we admit the possibility.

There is a certain degree of fetich worship in regard to this "cause" of disease. A dagger inserted in the heart causes a wound—removal of the dagger hardly cures it. A swarm of micro-organisms descends on a toxic patch of tissue in the lungs—removal of the intruders does not necessarily leave the lung intact. There is a failure to distinguish between the original cause of a malady and the results of that malady. Each demands treatment; each responds in a different manner to therapy. How long we bungled over cases of cerebral syphilis before we appreciated its truth, and learned that we must intervene as quickly and as powerfully as possible in order to prevent damage to the delicate tissues which not all the mercury out of Spain could restore! Remove the cause of disease, to be sure; treat the cause whenever you find it still acting, but don't limit your therapeutic activity to that one method or argue that there is no room for any other indication.

It is the all but universal presence of vasomotor disequilibrium in the whole wide field of clinical medicine that compels such constant reference to the four great vasomotor remedies in the practice of our art—aconitin, veratrin, digitalin, and strychnin. These, singly or combined, form an essential part of the therapy of so many cases of so many maladies that they, above all other remedies, are constantly mentioned in my work. That the causal indication is not neglected is shown by the interminable injunction to first clear the bowels and disinfect them, and to keep doing so, since observation has shown with what frequency the absorption of toxins from the alimentary canal coincides with the occurrence of disease in limited areas where the vital resistance is presumably lowest.

Whether autoinfection occasions disease directly from chemical action or, by still further lowering resistance, opens the door to invading microbes makes little difference—the indication for establishing intestinal exosmosis is similarly almost universal. The man who appreciates the importance of these two pathological principles, the fact of autotoxemia and the constant presence of vasomotor instability, has a broad foundation on which to build his practice. To this he can at every step add something—he never has to unlearn these two lessons. Making these the basis, never neglecting them in any case, we find that every patient displays indications for something more, but never with a frequency comparable with that of these two.

We constantly find the necessity of referring back to the newer works on physiology, and we glean every scrap of pathological information we can find, for we can not afford to be ignorant of a single fact that may explain our cases. So also we must possess hundreds of remedial agents, although nine-tenths of our work may be done with a score. But it is in the remaining tenth that we find the greatest pleasures of the practice of medicine, when by the exhibition of some definite active principle that has not been needed for years we meet an indication that has not presented in that period, but for which we have had the remedy waiting.

I beg my readers to pardon me for the importunity with which I beseech them to consider these truths. I can not help it; I do so want them to know of these exquisitely differentiated remedies that I can not help being urgent. And, knowing these things so well, how important they are for the profession and for suffering humanity, when I find that one does not really know there is a difference between hyoscine and atropine I am ready to lie down and die.—*Medical Record,* Nov. 10, 1906.

